# High Capacitance Density and Thermal Stability in Strontium

**DOI:** 10.3390/ma18081687

**Published:** 2025-04-08

**Authors:** Yilong Feng, Zhenya Lu, Ming Lv

**Affiliations:** School of Materials Science and Engineering, South China University of Technology, Guangzhou 510640, China; xionlong@163.com

**Keywords:** SrTiO_3_ thin films, RF magnetron sputtering, capacitance density, TCC

## Abstract

Magnetron sputtering allows for the accurate estimation of film thickness. Strontium titanate (STO) thin films were deposited on Nb-doped STO substrates using radiofrequency magnetron sputtering technology. The microstructures and dielectric properties of STO thin films were investigated. X-ray diffraction (XRD) analysis indicates that uniform polycrystalline STO films were obtained after thermal annealing at 650 °C. The films exhibit a significant correlation between thickness, annealing temperature, and breakdown field strength. The optimal film with a thickness of 1150 nm achieves a capacitance density of 1688 pF/mm^2^ and a breakdown field strength of 270 kV/mm. Additionally, STO films annealed at 650 °C maintained their capacitance value within ±15% across a temperature range of −55 °C to 125 °C. These results highlight the potential of STO thin films for high-performance capacitor applications.

## 1. Introduction

The rapid development of portable electronic devices and wearable technologies has highlighted the significance of miniaturization and weight reduction in electronic components. This trend has resulted in a substantial surge in market demand for capacitors that are compact, lightweight, and possess high-performance storage capabilities. These capacitors must efficiently store and release electrical energy within confined spaces to guarantee the prolonged and stable operation of devices. Additionally, they must satisfy the energy demands of high-performance applications, including rapid charging and high-power delivery.

In industrial settings, the continuous advancement of robotics and power systems has further fueled the demand for capacitors. Large-capacity capacitors play a pivotal role in these applications by stabilizing voltage fluctuations and providing instantaneous high-current support. This capability is crucial for maintaining the stability and responsiveness of robots during complex tasks. Consequently, the demand for capacitors in modern society is experiencing unprecedented growth, driven not only by the miniaturization of consumer electronics but also by a diverse range of industrial applications. As we explore the various applications of miniaturized capacitors, it is essential to shift our attention to capacitors, which hold a pivotal position in electronics. Their unique structure offers new performance and application possibilities, making the development of capacitors with high capacitance density increasingly urgent [1]. Ongoing advancements in artificial intelligence are further amplifying the need for enhanced storage capacity. In response, the power ratings of capacitors are being progressively improved [2].

Thin-film capacitors, recognized for their efficiency, are often silicon-based and utilize advanced dielectric materials such as SiO_2_ and STO [3]. For instance, the orthorhombic phase of perovskite-structured CaZrO_3_, when deposited on a Pt/Ti/SiO_2_/Si substrate, can achieve a dielectric constant as high as 39.42 [4]. Tumarkin et al. fabricated SrTiO_3_ thin films via RF magnetron sputtering, achieving high capacitance tunability (46%) and low loss (0.009–0.014) at 2 GHz [3]. Perovskite materials, known for their unique physical and chemical properties, offer significant advantages in energy storage applications. Compared to traditional energy storage technologies, such as lithium-ion batteries, perovskite batteries demonstrate higher energy density. Their efficient photoelectric conversion capabilities have already been validated in photovoltaic applications. Additionally, perovskite materials can effectively convert various forms of energy—such as light and thermal energy—into electrical energy for storage, catering to a wide range of applications, including residential energy storage, photovoltaic battery systems, and intelligent devices.

STO, equipped with high dielectric constant, low dielectric loss, and good thermal stability, has a perovskite structure. Thin-film technology is employed to leverage the performance benefits of STO materials, allowing precise control over parameters such as thickness and structure, thus enhancing their performance and application potential. Various methods for thin-film preparation include chemical vapor deposition [5], RF magnetron sputtering [6,7], pulsed laser deposition [8,9,10], and the sol–gel method [11,12]. RF magnetron sputtering technology enables the production of high-quality STO thin films through the precise control of sputtering conditions and material composition. These factors are crucial for improving the capacitance density, breakdown voltage, and reliability of electronic devices. Furlan et al. successfully synthesized carbon phosphide films via magnetron sputtering, showcasing the technology’s potential for creating non-traditional materials. Building on this approach, the present study utilizes RF magnetron sputtering to fabricate strontium titanate thin films exhibiting high capacitance density and excellent thermal stability [13]. Cecilia Goyenola et al. successfully synthesized CS_x_F_y_ films with tailored structures and properties through a combination of theoretical prediction and experimental synthesis. This work not only showcased the vast potential of magnetron sputtering technology in the fabrication of carbon-based films but also served as a valuable reference for the preparation of various other film types [14]. Moreover, a pivotal advantage of the sputtering technique lies in its inherent compatibility with existing thin-film circuit fabrication workflows. Given the widespread employment of sputter-deposited metal interconnects in high-performance capacitor components, the adoption of this method for STO thin-film capacitor fabrication enables inline monolithic integration. This process synergy not only mitigates the challenges associated with hybrid packaging but also significantly enhances component miniaturization and system-level integration efficiency, thereby fulfilling critical requirements for high-performance capacitors.

The choice of STO for both the film and substrate is based on the similarity of their lattice constants, which ensures excellent lattice matching. This matching reduces defects and mechanical stress [15], thereby enhancing the crystalline quality of the films. However, controlling the thickness of the STO thin film is crucial to avoid defects such as cracking due to internal stresses, an issue highlighted in the literature [15].

The electrical properties of capacitors are predominantly determined by the thickness and quality of the insulating film. This study focuses on optimizing capacitor performance by precisely controlling annealing conditions, as these parameters critically influence the thickness, density, and structural uniformity of the insulator.

To achieve this, STO thin films within capacitors were annealed across a range of temperatures, and their electrical characteristics—including capacitance, leakage current, and dielectric loss—were systematically analyzed. This approach elucidates the interplay between annealing parameters and film properties, providing insights into tailoring fabrication processes to enhance capacitor reliability and efficiency in advanced electronic applications.

## 2. Materials and Methods

### 2.1. Thin Film Preparation

**Figure 1:**
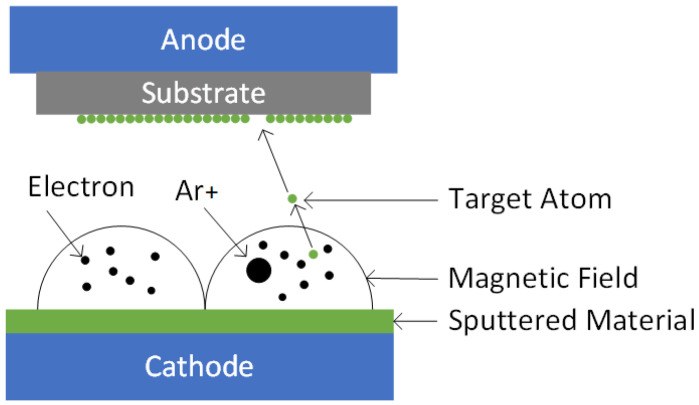
Sputtering coating schematic diagram.

The STO film was grown on the substrate using a radio frequency (RF) magnetron sputtering process (see Figure 1). For the experimental materials, single-crystal STO (n-type) doped with Nb sourced from Hefei Crystal & Optoelectronic Materials Co., Ltd. (Hefei, Anhui, China), was chosen as the substrate. It exhibited a resistivity of 0.007 Ω·cm and a surface roughness of less than 5 nm post-polishing, fulfilling the requirements for experimental accuracy. All substrates were cut in the (100) crystallographic direction. The optimized magnetron sputtering conditions, encompassing substrate temperature and power configuration, were determined through iterative literature correlation and experimental validation [3]. During the sputtering process, a mixture of argon and oxygen gasses, with a ratio of 80:2, was used as the sputtering atmosphere to optimize the deposition conditions. The sputtering parameters were meticulously controlled, with a temperature maintained at 250 °C and a power setting of 140 W, to ensure optimal sputtering results.

**Figure 2:**
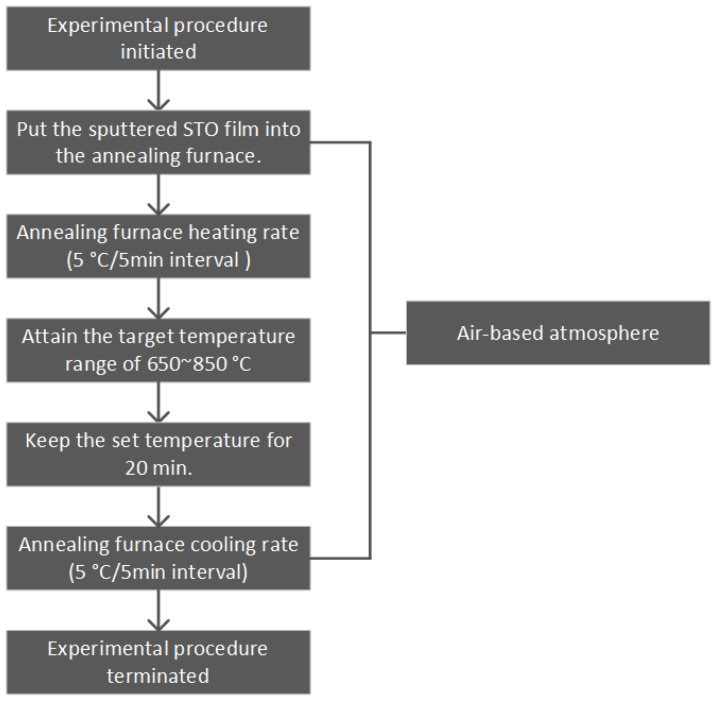
Annealing steps scheme.

After the sputtering process was completed, the thin film underwent an annealing treatment in an air atmosphere. The temperature was maintained within the range of 650 to 850 °C for a duration of 20 min. Both thermal cycles were controlled at a rate of 5 °C/5 min, ensuring homogeneous treatment conditions. The corresponding annealing temperature profile is illustrated in Figure 2. Subsequently, a circular gold electrode with a diameter of 100 μm was deposited onto the film’s surface using a mask, enabling the performance of electrical measurements.

### 2.2. Characterization of Thin Film Structure and Dielectric Properties

In this study, we used a polycrystalline XRD (Panalytic X’Pert Pro, Almelo, The Netherlands) to analyze the crystal structures of the samples. Simultaneously, a field emission scanning electron microscope (FESEM Quanta-400f, Eindhoven, The Netherlands) was utilized to observe the grain morphologies. The impedance properties of the samples were measured using an inductance, capacitance, and resistance meter (Agilent E4980A, Santa Clara, CA, USA). Additionally, an impedance analyzer (Keysight E4990A, Santa Clara, CA, USA) was employed to characterize the capacitance–voltage (C-V) and current–voltage (I-V) characteristics. Additionally, Electronic Component Burn-In Testers (EST100SLCBT, Wuxi, Jiangsu, China) were used for aging experiments.

## 3. Results and Discussion

**Figure 3:**
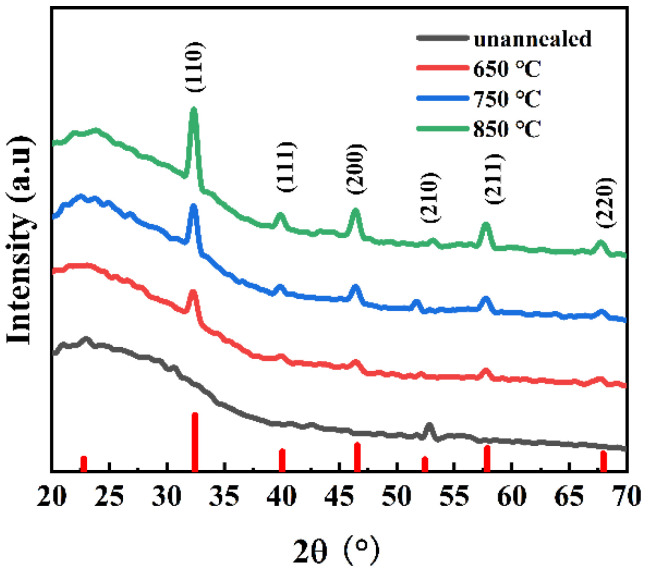
XRD patterns of STO.

Figure 3 presents the XRD patterns of perovskite STO thin films sputtered on single-crystal STO substrates, which were treated with different annealing temperatures to characterize their respective crystal structures. The XRD pattern of the unannealed STO film shows no detectable diffraction peaks, indicative of an amorphous structure. This suggests the presence of long-range disorder within the crystals and incomplete crystal formation. Following annealing, the diffraction peaks of the films become more pronounced, indicating the gradual formation of crystals exhibiting a cubic crystal system. Notably, the diffraction peaks of the STO films annealed at 650, 750, and 850 °C are significantly more prominent at the (110) crystal plane compared to other crystal planes, suggesting that crystal growth primarily occurs along the (110) direction. Furthermore, a marked increase in crystallinity is observed with increasing annealing temperature, confirming the influence of annealing temperature on the growth of STO crystals. This suggests that annealing enhances the interaction between the internal components of the freshly deposited films, contributing positively to the film preparation process [16].

**Figure 4:**
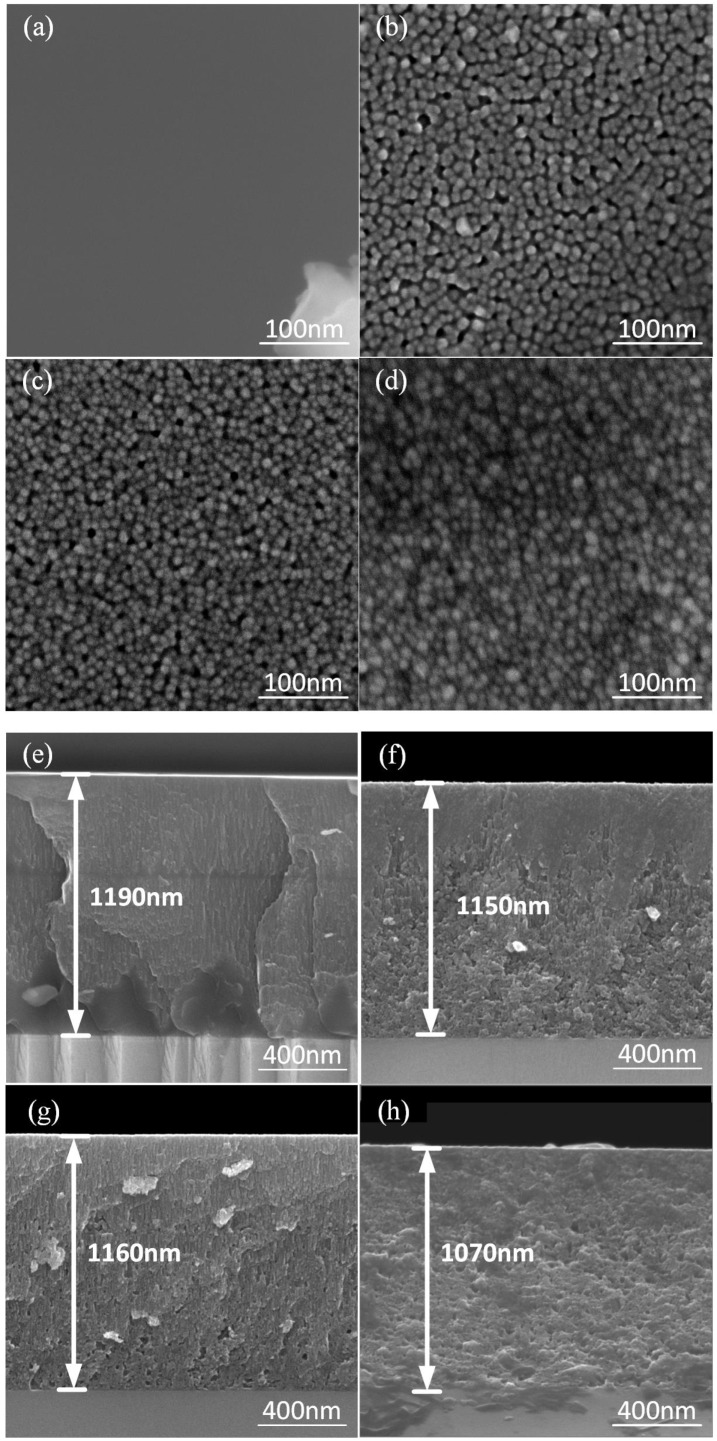
SEM images of STO thin films’ surface (**a**–**d**) and cross-section (**e**,**f**) at different annealing temperatures: (**a**,**e**) unannealed, (**b**,**f**) annealed at 650 °C, (**c**,**g**) annealed at 750 °C, and (**d**,**h**) annealed at 850 °C.

Figure 4 illustrates the surface morphologies of STO thin films observed via SEM after exposure to various annealing temperatures. In the absence of annealing, as depicted in Figure 4a,e, the film exhibits an amorphous structure characterized by a higher internal energy state. Annealing is necessary to convert the film into a crystalline phase, thereby reducing internal energy and increasing stability. As shown in Figure 4b, annealing at 650 °C results in the formation of fine, densely packed surface grains, likely due to reduced thermal expansion mismatches between the STO film and the single-crystal STO substrate. This improved film density is expected to enhance its electrical performance. It can be observed that the maximum thickness of the uniform STO thin film sputtered onto the single-crystal substrate is 1150 nm [15], which is greater than the 600 nm thickness achieved when sputtered directly onto a silicon substrate. As the annealing temperature increases, the number of gaps between the grains gradually increases. At 850 °C, as shown in Figure 4d, the voids between the particles on the film’s surface become larger and more numerous. Morphological changes are evident in Figure 4h. At higher annealing temperatures (850 °C), grains undergo significant growth, leading to lattice distortions and the formation or exacerbation of defects such as dislocations and grain boundaries [17]. These defects can act as obstacles to the movement of charge carriers, resulting in reduced mobility [18].

Table 1 summarizes the electrical properties of STO thin films annealed at different temperatures. To systematically evaluate the effect of annealing temperature on electrical performance, we characterized four sample sets with combined XRD crystallinity analysis and surface morphology observations. The results demonstrate a strong correlation between optimized crystal structure and enhanced electrical properties (Table 1). All electrical measurements were conducted in rigorously controlled conditions to ensure comparability: a 0.1 mm diameter electrode was used to maintain a uniform current flow area, while capacitance, dielectric loss, and dielectric constant measurements were performed at 1 MHz to probe high-frequency response characteristics. Insulation resistance was tested at 50 V to evaluate dielectric strength. This revealed that annealing temperature critically modulates the electrical behavior of STO thin films, with optimal performance achieved at 650 °C.

Table 1 shows that the thin film annealed at 650 °C exhibited a capacitance of 53 pF, and the lowest dielectric loss was measured at 49 × 10^−4^. Compared with the unannealed STO thin films, it is reasonable to speculate that a certain degree of high-temperature annealing may result in surface reconstruction or local distortions of the films, subsequently leading to an increase in capacitance. This sample also demonstrated the highest insulation resistance, recorded at 1036 GΩ. The permittivity of this film was the highest among the samples, reaching 220 at a test frequency of 1 MHz. Furthermore, the breakdown voltage of the thin films varied with annealing temperature; the film annealed at 650 °C had the highest breakdown voltage of 310 V. As depicted in Figure 4b, the void density in the film was significantly reduced when annealed at 650 °C, which likely accounts for the observed increase in breakdown voltage [19]. According to Equation (1), the breakdown field strength was calculated to be 270 kV/mm.

E = U/d,(1)

Here, E denotes the electric field strength in kV/mm, U represents the applied voltage in V, and d signifies the sample thickness in mm. These data are essential for analyzing the electrical behavior of STO thin films at various annealing temperatures and will contribute to future research on capacitor devices.

**Figure 5:**
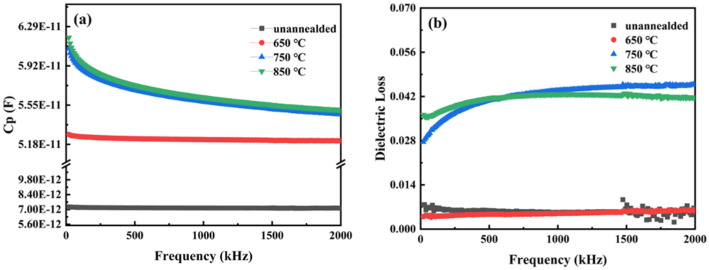
Frequency dependence of capacitance for STO thin films subjected to various annealing treatments. (**a**) relationship of STO film capacitance with frequency at different annealing temperatures. (**b**) relationship of STO film dielectric loss with frequency at different annealing temperatures.

Figure 5 illustrates the relationship between capacitance and frequency of STO thin films after various annealing treatments, which is essential for understanding the electrical behavior of STO thin films in practical applications. In the design and application of a capacitor, it is crucial to maintain capacitance variation with frequencies within specified limits to ensure the consistent performance of circuits and systems [20,21,22]. As shown in Figure 5, both the unannealed STO thin films and those annealed at 650 °C exhibit relatively stable capacitance values within the frequency range of 0–2 MHz. Additionally, under the annealing condition of 650 °C, the capacitance remains stable at 5.21 × 10^−11^ F, while the dielectric loss remains low at approximately 4.5 × 10^−3^. This stability in capacitance with frequency indicates the STO thin films’ reliable electrical performance across a wide frequency range. Firstly, this stability ensures the consistency and predictability of STO thin films under varying frequency operating conditions, which is critical for high-performance capacitors. STO thin films can sustain a stable capacitance value, minimizing performance variations due to frequency changes and thus enhancing system stability and efficiency. Secondly, the stability of capacitance with frequency correlates with the stability of the dielectric constant and dielectric loss of STO thin films. As presented in Table 1, after annealing at 650 °C, STO thin films exhibit high capacitance density (1688 pF/mm^2^), low dielectric loss (49 × 10^−4^), and maintain a relatively stable dielectric constant (220) within the tested frequency range. This stability in dielectric properties further supports the reliability of STO thin films across different application conditions, making them suitable for high-performance capacitors [23].

**Figure 6:**
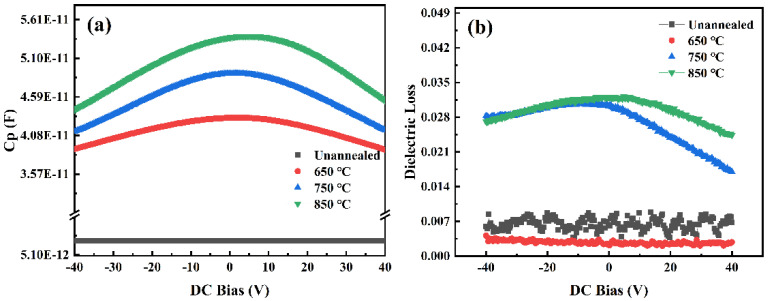
C-V characteristics of STO thin film capacitors under applied bias voltage following different annealing treatments. (**a**) relationship of STO film capacitance with DC bias at different annealing temperatures. (**b**) relationship of STO film dielectric loss with DC bias at different annealing temperatures.

Figure 6 displays the capacitance–voltage (C-V) curve of a typical STO thin film at room temperature. C-V characteristics are crucial for analyzing capacitors’ electrical properties. Typically, small-signal, high-frequency C-V measurement techniques are used to capture these characteristics accurately. Ideally, capacitance remains constant across frequencies, but in practice, charge accumulation and discharge occur under forward bias, leading to various phenomena in the device. The electrical characteristics of the STO thin film were evaluated through the measurement of its C-V characteristics at the same temperature. During measurement, the bias voltage was swept from −40 V to 0 V and then ramped up to 40 V, allowing us to observe the variations in capacitance with voltage. The scan rate was maintained at 1 V/s to ensure measurement accuracy and stability. The C-V curve demonstrates that as the bias voltage gradually increases, the capacitance value of the annealed STO thin film exhibits a general downward trend. This observation can be explained by the complex variations in charge distribution and electric field response within the STO thin film due to voltage changes [24]. Specifically, an increase in bias voltage enhances the electric field intensity within the film, potentially altering the charge distribution, which subsequently affects its capacitance value [25]. Additionally, the annealing process likely influences the microstructure and electrical properties of the STO thin film, further modifying its C-V characteristics.

Analyzing the C-V curve offers insights into the electrical behavior of STO thin films and the impact of annealing treatment on their performance. The observed behavior can be explained by changes in internal charge distribution, defect formation, or the redistribution of the electric field within the film [24]. At an annealing temperature of 650 °C, the capacitance of the STO thin film measured approximately 3.82 × 10^−11^ F. Notably, the dielectric loss observed at this temperature was the lowest compared to the other two annealing temperatures investigated. These results suggest that bias voltage significantly impacts the capacitance characteristics of STO thin films, providing insights into their electrical behavior [26,27,28].

**Figure 7:**
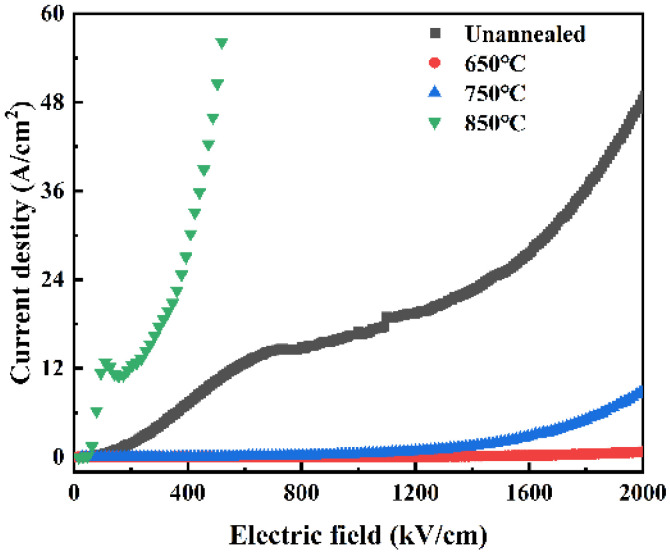
I-V characteristics of STO thin films with varying electrode areas following different annealing treatments.

Figure 7 illustrates the impact of various annealing treatments on the insulating properties of STO thin films. Leakage current I-V testing of thin films is crucial for assessing performance, detecting defects, estimating stability, and refining device design [29,30,31]. As shown in Figure 7, the analysis of the I-V curves at different annealing temperatures reveals that the STO film annealed at 650 °C exhibits the lowest leakage current. This observation suggests that at this specific temperature, the crystalline structure of the material undergoes significant improvement, leading to an overall enhancement in the quality of the film. This optimization results in a reduction in internal defects and, consequently, a marked improvement in the insulating properties of the material. These changes are evident in the I-V curves, characterized by a reduction in leakage current. After annealing at 650 °C, the maximum current density remains low with a radius of 100 μm, indicating the beneficial effect of annealing on insulation performance. As the annealing temperature increases further, a noticeable rise in leakage current is observed. It has been demonstrated that as temperature increases, the leakage current in ceramic materials can rise significantly, compromising their overall performance. Xie et al. discussed the resistivity degradation of strontium titanate-based ceramics, highlighting that high temperatures facilitate the thermal excitation of charge carriers, subsequently leading to an increase in leakage current [32]. This could be attributed to excessive crystallization or thermal stress caused by high-temperature annealing, leading to the creation of new defects or cracks within the film that facilitate additional leakage paths for charge carriers. These findings indicate that STO films subjected to annealing at 650 °C exhibit promising application prospects and offer insightful guidance for high-performance capacitors.

**Figure 8:**
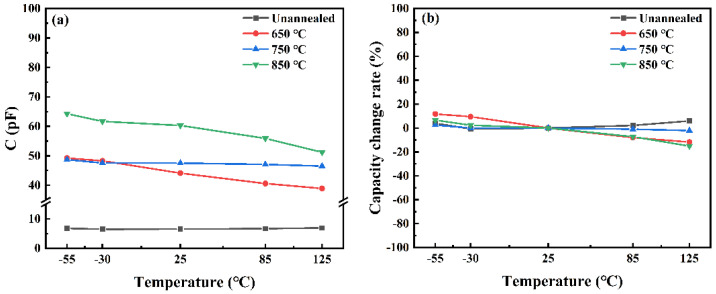
Temperature coefficient of capacitance (TCC) of STO thin films undergoing different annealing processes. (**a**) temperature-dependent capacitance of STO films annealed at various temperatures. (**b**) temperature-dependent dielectric loss of STO films annealed at various temperatures.

Figure 8 shows the TCC of STO films annealed at various temperatures, evaluating their capacitance stability under different thermal conditions. For high-performance capacitors, consistent performance across temperatures is essential. From Figure 8, it can be observed that the capacitance of the unannealed STO thin film remains almost constant with temperature changes, yet its capacitance value is quite low. After annealing, the capacitance values are higher than those of the unannealed film, demonstrating that annealing can effectively enhance the capacitance of the STO thin film. Specifically, under the 650 °C annealing condition, the STO film demonstrates superior capacitance stability, with a capacitance variation rate within ±15% over the entire test temperature range. Under the 850 °C annealing condition, although the film’s capacitance density increases, its TCC curve exhibits a downward trend at higher temperatures, possibly due to lattice distortion or phase transition. Conversely, excessively high annealing temperatures, such as 850 °C, may lead to the formation of new thermal stresses or defects [33], resulting in a slight decline in capacitance temperature stability. In conclusion, the data presented in Figure 8 indicate that 650 °C is the optimal annealing temperature for enhancing the TCC performance of STO films. Films annealed at this temperature not only exhibit high capacitance density and breakdown field strength but also demonstrate excellent capacitance temperature stability.

As shown in Table 2, all the data presented are electrical properties measured after the aging experiments (100 V, 125 °C, 500 h). Aging tests on capacitive products are essential for enhancing reliability, assessing service life, optimizing design, ensuring compliance, reducing costs, and safeguarding safety. By simulating long-term conditions, potential issues are identified early, ensuring stability and reliability in practical use. As can be seen from Table 2, the capacitance density of the STO thin film annealed at 650 °C remains at 1592 pF/mm^2^, which is only a 5.68% decrease from the pre-aging value of 1688 pF/mm^2^. This relatively low rate of change in capacitance density is highly beneficial for the practical application of capacitors.

## 4. Summary and Conclusions

In summary, this study demonstrates that by carefully adjusting the annealing temperature and effectively utilizing the magnetron sputtering thin-film process, the perovskite STO phase can be engineered effectively. The annealing temperature plays a critical role in phase formation: STO films annealed at 650 °C exhibit a capacitance density of 1688 pF/mm^2^ under an electric field strength of 270 kV/mm. XRD analysis confirms polycrystalline uniformity, indicating the potential for reproducible large-area device fabrication, which is essential for scaling capacitor production in grid-scale energy storage applications. This is essential for scaling capacitor production in grid-scale energy storage applications. The capacitance that remains stable over a temperature range of −55 °C to 125 °C meets the operational requirements of automotive electronics and spacecraft avionics. Furthermore, as evidenced by XRD, the correlation between annealing temperature and microstructure quality provides a design strategy to optimize dielectric permittivity in STO-based tunable microwave devices, such as 5G mobile-network phase shifters. In addition, the 650 °C annealed film of this work has a relatively low dielectric loss of 4.9 × 10^−3^ and relatively high dielectric constant of 220 (Table 1). Consequently, this work advances the performance of high-performance capacitors for next-generation electronic systems.

## Figures and Tables

**Table 1:** Influence of annealing temperature on the electrical properties of STO thin films.

Annealing Temperature (°C)	Thickness (nm)	C (pF)	D (×10^−4^)	I.R (GΩ)	Capacitance Density (pF/mm^2^)	Breakdown Voltage (V)	Breakdown Field Strength (kV/mm)	Dielectric Constant
Unannealed	1190	7	121	154	223	349	293	30
650	1150	53	49	1036	1688	310	270	220
750	1160	52	451	471	1656	210	181	217
850	1070	59	419	380	1879	150	140	227

**Table 2:** Influence of aging test on the electrical properties of STO thin films.

Annealing Temperature (°C)	Thickness (nm)	C (pF)	D (×10^−4^)	Capacitance Density (pF/mm^2^)	Rate of Change of Capacitance Density (%)
Unannealed	1190	10	159	318	42.6
650	1150	50	84	1592	−5.68
750	1160	49	490	1560	−7.28
850	1070	55	430	1751	−6.8

## Data Availability

The original contributions presented in this study are included in the article. Further inquiries can be directed to the corresponding author.

## References

[1] Molas G., Nowak E. (2021). Advances in Emerging Memory Technologies: From Data Storage to Artificial Intelligence. Appl. Sci..

[2] Li X., Zhang X., Lin F., Blaabjerg F. (2021). Artificial-Intelligence-Based Design for Circuit Parameters of Power Converters. IEEE Trans. Ind. Electron..

[3] Tumarkin A., Sapego E., Gagarin A., Bogdan A., Karamov A., Serenkov I., Sakharov V. (2023). SrTiO_3_ Thin Films on Dielectric Substrates for Microwave Applications. Coatings.

[4] Zhao C., Wang Y., Li Z., Chen W., Xu Q., He D., Xi D., Zhang Q., Yuan T., Qu Y. (2019). Solid-Diffusion Synthesis of Single-Atom Catalysts Directly from Bulk Metal for Efficient CO_2_ Reduction. Joule.

[5] Kon D., Hashiba K., Kawashima T., Akiyama Y. (2009). Preparation of Strontium Titanate Film by Low Pressure Chemical Vapor Deposition. ECS Trans..

[6] Karaki T., Du J., Fujii T., Adachi M. (2002). Electrical Properties of Epitaxial (Pb,Sr)TiO_3_ Thin Films Prepared by RF Magnetron Sputtering. Jpn. J. Appl. Phys..

[7] Wang Y.-H., Rahman K.H., Wu C.-C., Chen K.-C. (2020). A Review on the Pathways of the Improved Structural Characteristics and Photocatalytic Performance of Titanium Dioxide (TiO_2_) Thin Films Fabricated by the Magnetron-Sputtering Technique. Catalysts.

[8] Marozau I., Shkabko A., Dinescu G., Döbeli M., Lippert T., Logvinovich D., Mallepell M., Weidenkaff A., Wokaun A. (2008). RF-plasma assisted pulsed laser deposition of nitrogen-doped SrTiO_3_ thin films. Appl. Phys. A.

[9] Shan F.K., Shin B.C., Jang S.W., Yu Y.S. (2004). Substrate effects of ZnO thin films prepared by PLD technique. J. Eur. Ceram. Soc..

[10] Venkatachalam S., Iida Y., Kanno Y. (2008). Preparation and characterization of Al doped ZnO thin films by PLD. Superlattices Microstruct..

[11] Mansilla Y., Arce M.D., González-Oliver C., Basbus J., Troiani H., Serquis A. (2021). Characterization of stabilized ZrO_2_ thin films obtained by sol-gel method. Appl. Surf. Sci..

[12] Cao Y., Li J., Li X., Xie Y., Feng Q., Zhang X., Tuo X. (2024). A sol-gel strategy for constructing a hydroxyapatite nanowire/aramid nanofiber electric insulating composite with excellent flame retardancy and mechanical property. Mater. Today Commun..

[13] Furlan A., Gueorguiev G.K., Czigány Z., Högberg H., Braun S., Stafström S., Hultman L. (2008). Synthesis of phosphorus-carbide thin films by magnetron sputtering. Phys. Status Solidi (RRL)-Rapid Res. Lett..

[14] Goyenola C., Lai C.-C., Näslund L.-Å., Lu J., Högberg H., Hultman L., Rosen J., Gueorguiev G.K. (2016). Theoretical Prediction and Synthesis of CSxFy Thin Films. J. Phys. Chem. C.

[15] Deshmukh V.V., Ravikumar C.R., Kumar M.R.A., Ghotekar S., Kumar A.N., Jahagirdar A.A., Murthy H.C.A. (2021). Structure, morphology and electrochemical properties of SrTiO_3_ perovskite: Photocatalytic and supercapacitor applications. Environ. Chem. Ecotoxicol..

[16] Khan S.B., Zhang Z., Lee S.L. (2020). Annealing influence on optical performance of HfO_2_ thin films. J. Alloys Compd..

[17] Zhang Y.-Q., Quan G.-Z., Zhao J., Yu Y.-Z., Xiong W. (2023). A Review on Controlling Grain Boundary Character Distribution during Twinning-Related Grain Boundary Engineering of Face-Centered Cubic Materials. Materials.

[18] Tailor N.K., Yukta, Ranjan R., Ranjan S., Sharma T., Singh A., Garg A., Nalwa K.S., Gupta R.K., Satapathi S. (2021). The effect of dimensionality on the charge carrier mobility of halide perovskites. J. Mater. Chem. A.

[19] Baumert B.A., Chang L.-H., Matsuda A.T., Tsai T.-L., Tracy C.J., Gregory R.B., Fejes P.L., Cave N.G., Chen W., Taylor D.J. (1997). Characterization of sputtered barium strontium titanate and strontium titanate-thin films. J. Appl. Phys..

[20] He Q., Sun K., Shi Z., Liu Y., Fan R. (2023). Polymer dielectrics for capacitive energy storage: From theories, materials to industrial capacitors. Mater. Today.

[21] Kaur R., Arora A., Tripathi S.K. (2020). Fabrication and characterization of metal insulator semiconductor Ag/PVA/GO/PVA/n-Si/Ag device. Microelectron. Eng..

[22] Zhao S., Fang J., Wang Y., Zhang Y., Zhou Y., Zhuo S. (2020). Construction of three-dimensional mesoporous carbon nitride with high surface area for efficient visible-light-driven hydrogen evolution. J. Colloid Interface Sci..

[23] Hou C., Huang W., Zhao W., Zhang D., Yin Y., Li X. (2017). Ultrahigh Energy Density in SrTiO_3_ Film Capacitors. ACS Appl. Mater. Interfaces.

[24] Kim D., Lee G., Seo M., Cha B., Lee C., Ahn Y.G., Kim H.-S. (2024). Experimental evaluation of saturation capacitance in aging phenomena in multi-layer ceramic capacitors (MLCCs). Ceram. Int..

[25] Fuchs D., Schneider C.W., Schneider R., Rietschel H. (1999). High dielectric constant and tunability of epitaxial SrTiO_3_ thin film capacitors. J. Appl. Phys..

[26] Zeng X.K., Li Y.T., Zhang X.D., Liu M., Ye J.Z., Qiu X.L., Jiang X., Leng Y.X. (2023). Effect of bias voltage on the structure and properties of CuNiTiNbCr dual-phase high entropy alloy films. J. Alloys Compd..

[27] Sun L., Li H., Wei N., Li J., Huang J., Kong J., Wu Q., Shi Y., Xiong D. (2024). Effects of bias voltage on the structure, mechanical properties and tribological properties of TaBx films at elevated temperatures. Int. J. Refract. Met. Hard Mater..

[28] Khaldi O., Jomni F., Gonon P., Vallée C. (2020). AC and DC bias effect on capacitance–voltage nonlinearities in Au/HfO_2_/M (M = Pt, TiN, W, and AlCu) MIM capacitors: Effect of the bottom electrode material. J. Mater. Sci. Mater. Electron..

[29] Tomilin S.V., Yanovsky A.S., Tomilina O.A., Mikaelyan G.R. (2013). Study of the I–V characteristics of nanostructured Pd films on a Si substrate after vacuum annealing. Semiconductors.

[30] Kraidy A.F., El Radaf I.M., Zeinert A., Lahmar A., Pelaiz-Barranco A., Gagou Y. (2024). Optoelectrical properties of the ternary chalcogenide SnSb_2_S_5_ as a new absorber layer for photovoltaic application. J. Phys. D Appl. Phys..

[31] Shi N., Lv Y., Zhang Y., Zhu X. (2023). Linear fitting Rule of I–V characteristics of thin-film cells based on Bezier function. Energy.

[32] Xie H., Pu Y., Shang Y., Zhang L., Wang B., Hao Y. (2023). Suppressing resistance degradation in SrTiO_3_-based colossal permittivity capacitor material. Ceram. Int..

[33] Umran H.M., Wang F., He Y. (2020). Ageing: Causes and Effects on the Reliability of Polypropylene Film Used for HVDC Capacitor. IEEE Access.

